# Developmental pluripotency-associated 4: a novel predictor for prognosis and a potential therapeutic target for colon cancer

**DOI:** 10.1186/s13046-015-0176-z

**Published:** 2015-06-11

**Authors:** Meng Zhang, Feifei Cui, Su Lu, Huijun Lu, Yingming Xue, Jingtao Wang, Jian Chen, Senlin Zhao, Shaofei Ma, Yu Zhang, Yang Yu, Zhihai Peng, Huamei Tang

**Affiliations:** Departments of Pathology, Shanghai Jiaotong University Affiiated First People’s Hospital, Shanghai, 200080 People’s Republic of China; Departments of General Surgery, Shanghai Jiaotong University Affiiated First People’s Hospital, Shanghai, 200080 People’s Republic of China

**Keywords:** Colon cancer, Dppa4, Immunohistochemistry, Prognosis, Proliferation

## Abstract

**Backgrounds:**

Developmental pluripotency-associated 4 (Dppa4) gene plays an important role in self-renewal and pluripotency sustainability in embryonic stem cells. It is re-expressed in several malignant tumors and is identified as a new pluripotency-related oncogene. The present study investigates the expression and clinical significance of Dppa4 in colon cancer.

**Methods:**

Real-time polymerase chain reaction and Western blotting were used to evaluate Dppa4 mRNA and protein expression in 39 pairs of fresh-frozzen colon cancer samples, which were compared with adjacent normal mucosa. The Dppa4 protein was evaluated by immunohistochemical techniques using colon tissue microarrays (TMA). The sample included 185 cancer specimens and corresponding normal colorectal mucosa. The effect of Dppa4 knockdown on colorectal cancer cell proliferation was investigated using Cell Counting Kit-8 (CCK8) assays and colony-formation assays.

**Results:**

Both the mRNA and protein level expression of Dppa4 gene was found to be upregulated in colon cancer tissues. Furthermore, the upregulated expression of Dppa4 was significantly correlated with the results of American Joint Committee on Cancer (AJCC) stage (*P* = 0.01), invasion depth (*P* = 0.028), nodal involvement (*P* = 0.012), distant metastasis (*P* = 0.003), and differentiation (*P* = 0.002). Dppa4 was also shown to be an independent prognostic indicator of disease-free survival (HR 6.118, 95 % CI 3.004–12.462) and overall survival (HR 6.348, 95 % CI 2.875–14.014) for patients with colon cancer. Knockdown of Dppa4 expression inhibited the proliferation of colorectal cancer cell lines through G1/S transition regulation.

**Conclusion:**

The results indicate that Dppa4 might play an important role in colon cancer progression and function as a novel prognostic indicator and a potential therapeutic target.

## Background

The relationship between cancer and stem cells is very astonishing [1]. Recent research supports the idea that human cancers can be considered as a stem cell disease [2]. The cancer-initiating cells, also known as cancer stem cells (CSCs), play an important role in hematopoietic cancers as well as in several solid tumors, including colon cancer [3–5]. According to the CSC hypothesis, cancer cells with stem cell-like features such as self-renewal and pluripotency sustains to have tumorigenic capacity [6, 7]. It seems that the molecular pathway of self-renewal in normal stem cells is the same as that of CSCs in tumor [8]. Many self-renewal regulatory factors, such as Oct4, Sox2, Bmi, and Nanog, are re-expressed in human malignant tumors, and they play an important role in carcinogenesis [9, 10].

Developmental pluripotency-associated 4 (Dppa4) is one of the uncharacterized genes that is highly expressed in embryonic stem (ES) cells. It has been reported that the expression of Dppa4 might be regulated by Oct4 and Sox2 genes [11] and Dppa4 could modulate chromatin structure in association with DNA and histone H3 in ES cells [12]. In a study of expression of stem cell-specific marker genes in multiple malignant tumors, Dppa4 was found to be significantly expressed in cancer cell lines and cancer tissues, including colon cancer and colorectal cell lines [9]. Po-Yuan Tung [13] *et al.* found that Dppa4 is an oncogene in both mouse 3 T3 cells and immortalized human dermal fibroblasts (HDFs). In addition, DPPA4 over-expression induces cell proliferation via genes related to the regulation of G1/S transition. However, the clinicopathological significance of Dppa4, and its possible mechanism in colon cancer tumorigenesis and progression is still unclear.

The current study was aimed to access the expression of Dppa4 at both transcriptional and translational levels and its predictive value in colon cancer. We investigated whether Dppa4 is an independent biomarker that could predict metastasis and prognosis in patients with colon cancer. By knockdown of Dppa4 expression with shRNA, we also investigated the effects of Dppa4 on colon cancer proliferation and its possible mechanisms.

## Materials and methods

### Patients and specimens

The study was conducted according to the Declaration of Helsinki and approved by the Ethics Committee of Shanghai Jiaotong University affiliated Shanghai First People’s Hospital Medical Center. Written informed consent was obtained from all patients. A total of 185 pairs of samples were obtained from patients with primary colon cancer who had undergone surgery without any preoperative therapy at the Shanghai Jiaotong University affiliated Shanghai First People’s Hospital Gastrointestinal Cancer Center between January 2001 and December 2003. Fresh samples were divided into two aliquots: One was snap-frozen into RNA Keeper Tissue Stabilizer (Vazyme Biotech Co., Ltd, Jiangsu, China), stored at 4 °C overnight, and then transferred to −80 °C for long-term storage; and the other was paraffin embedded for TMA construction. The diagnosis was confirmed by at least two pathologists. The anatomical location of the primary tumor was categorized as right, transverse, left, or sigmoid. Staging was based on pathological findings according to the American Joint Committee on Cancer (AJCC).

### TMA construction and immunohistochemistry

TMA construction was undertaken as reported previously [14]. Expression of Dppa4 was tested using standard immunohistochemical methods [15, 16]. The corresponding primary antibodies used were as follows, Dppa4 (1:200 dilution, ABGENT, San Diego, CA) and Ki-67 (1:50 dilution, Abcam, Cambridge, UK).

Based on the intensity and extent of staining the immunohistochemically stained slides were reevaluated by two independent observers who were blinded to patient information [17]. Briefly, Dppa4 cytoplasmic staining of the invasive tumor cells was designated with an intensity score (0 [no staining], 1 [weak staining], 2 [moderate staining], and 3 [strong staining]) and an extent score (0 [no staining of cells], 1 [<10 % of tissue stained positive], 2 [10 %–50 % stained positive], 3 [>50 % stained positive]). The intensity and extent score were then summed up to give a total score ranging from 0 to 6, with a total score of 0 to 2, 3 to 4, and 5 to 6 defined as no expression, weak expression, and strong expression of Dppa4. The Ki-67 proliferation index was based on the percentage of cells with positive nuclear staining and was divided into two groups: negative (≤10 % of cells with positive nuclei) and positive (>10 % of cells with positive nuclei).

### Cell culture and reagents

Human colon cancer cell lines RKO, LoVo, HCT116, SW480, SW620, HCT8, HT29, CaCo-2 were obtained from the Type Culture Collection of the Chinese Academy of Science (Shanghai, China) and cultured in Dulbecco’s modified Eagle’s medium (DMEM, Hyclone, Logan, UT), supplemented with 10 % fetal bovine serum (FBS, Gibco, Australia) and 1 % penicillin-streptomycin (Gibco,) in a humidified atmosphere containing 5 % CO_2_ at 37 °C.

### Dppa4 knockdown plasmid construction and cell transfection

To generate an effective Dppa4-shRNA oligonucleotide for gene knockdown studies, 4 Dppa4-shRNAs were designed and synthesized. The shRNA with the sequence 5’-GCAAAGTTCTGAGACACAT-3’ was determined to be the most effective shRNA in inhibiting Dppa4 expression. The non-target shRNA sequence 5’-TTCTCCGAACGTGTCACGT-3’ was employed as the negative control. The specific double-stranded oligonucleotides were chemosynthesized, annealed, and then inserted into a shRNA expression vector, GV248 (in collaboration with Shanghai GeneChem Company, China). The Dppa4-shRNA and negative control shRNA plasmids were transfected into Caco2 and HCT8 cells using Lipofectamine 2000 (Life Technologies, Rockville, MD). The Stable transfected cell clones were selected in 2lg/ml puromycin-containing medium (Sigma-Aldrich, St. Louis, MO). Dppa4 clone expressions were confirmed by qPCR and Western blot analysis.

### Cell proliferation assay and colony formation assay

The effect of Dppa4 knockdown on colon cancer cell proliferation was determined by measuring the absorbance at 450 nm. Briefly, untreated cells, cells treated with shRNA or non-target shRNA were re-suspended and seeded into 96-well plates (2 × 10^3^ cells/well) in triplicate. Cell counting kit-8 (CCK-8, Dojindo Co., Kumamoto, Japan) was added (10 μl) at 24, 48, and 72 h post-seeding. The absorbance at 450 nm was measured 2 h later.

Colony-formation ability was evaluated by preparing single-cell suspension solutions and then plating on a 6-well plate with 500 cells per well. After 14 days, the developed colonies were stained with Gimesa for 20 min, which were then counted and photographed. The tests were independently performed in triplicates.

### RNA extraction and real-time quantitative PCR

Total RNA was extracted from frozen tissue samples or cultured cells using Trizol (Invitrogen Life Technologies, Carlsbad, CA). After RNA intensity and purity was checked, 3 μg of total RNA was reverse-transcribed into the first strand of cDNA using the PrimeScript RT reagent kit (Takara, Shiga, Japan). Then 1 μl cDNA was used as a template for quantitative real-time PCR with SYBR Premix Ex Taq II (Takara, Shiga, Japan) according to manufacturer’s instructions. Below mentioned are the specific primers used: Dppa4, sense 5’- GACACAGATGGTTGGGTTCA-3’ and antisense 5’-GAGGCAGGAAGCAAGAAGAG-3’; GAPDH, sense 5’- AGAAGGCTGGGGCTCATTTG-3’ and antisense 5’-AGGGGCCATCCACAGTCTTC-3’; Ki67, sense 5’-TTCGCAAGCGCATAACCCA-3’ and antisense 5’-AACCGTGTCACAGTGCCAAA-3’; CCND1, sense 5’-GCTGCGAAGTGGAAACCATC-3’ and antisense 5’-CCTCCTTCTGCACACATTTGAA-3’; TAF10 sense 5’-CATTATCATGGGCCGACTCAAT-3’, antisense 5’-TGTGTAATCCTCCAACTGCATC-3’. GAPDH was used as the internal control. Each reaction was performed in triplicates. The relative DPPA4 mRNA expression was calculated by 2^-ΔΔCt^ comparative method.

### Western blot analysis

Total protein was isolated from tissue samples or cultured cells using RIPA lysis buffer and then quantified by the BCA assay kit (Beyotime Biotechnology, Jiangsu, China). Each 30 μg total protein was electrophoresed on 10 % SDS-polyacrylamide gel and then transferred to polyvinylidene fluoride (PVDF) membranes (Millipore, Billerica, MA). These membranes were blocked using 5 % non-fat milk with 0.1 % Tween 20 at room temperature for 1 h, followed by incubation with the appropriate primary antibodies: Dppa4 (1:500 dilution, ABGENT, San Diego, CA) and β-actin (1:1000 dilution, Abcam, Cambridge, UK) at 4 °C overnight. Following incubation with a horseradish peroxidase-conjugated secondary antibody (1:2000 dilution, Santa Cruz Biotechnology, USA), the blots were visualized by Immobilon Western Chemiluminescent HRP Substrate (Millipore, Billerica, MA) according to the manufacturer’s instructions. β-actin was used as the loading control. Each sample was loaded in triplicate.

### Statistical analysis

The differences between two groups were compared with the *t* test, *χ*^2^ test, or Fisher’s exact test, as appropriate. The patients’ survival curve was analyzed using the Kaplan-Meier method with the log-rank test. A Cox proportional hazards model was used to calculate univariate and multivariate hazard ratios for the variables. All analyses were carried out using the SPSS 19.0 software (SPSS Inc., Chicago, IL). All statistical analyses were set to a significance level of 0.05.

## Results

### Clinical information

Specimens were collected from 185 patients (79 men and 106 women) with an average age of 65.82 years (range, 22–95 years). Patient follow-up was carried out for 177 patients who had undergone curative operations according to the National Comprehensive Cancer Network Practice Guidelines in colon cancer (Engstrom PF.2005; 3:468-91). Disease-free survival (DFS) and overall survival (OS) rates were confronting each other, as per the interval from the initial surgery to clinically or radiologically proven recurrence/metastasis and death, respectively. The final follow-up was on June 29, 2008, with a median patient follow-up time of 60 months (range, 10–89 months). At the last follow up, 127 patients were still alive, whereas 50 had died, and 64 of the 177 patients experienced recurrence.

### Dppa4 expression and activity in human colon cancer

**Fig. 1:**
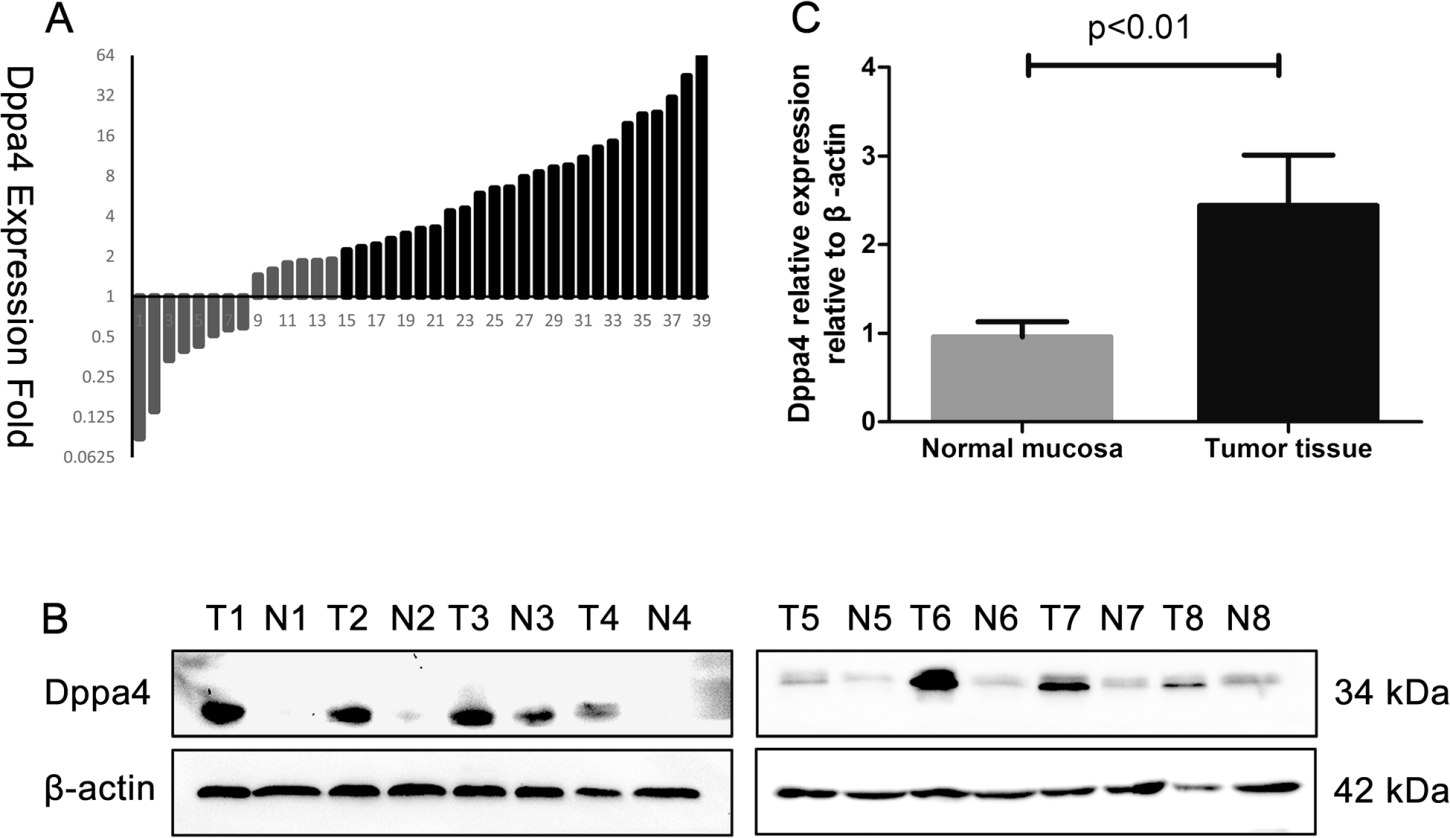
Analysis of Dppa4 expression in human colon cancer tissues. **a** Dppa4 mRNA levels in 39 primary tumor tissues and paired normal tissues detected by real-time PCR. Dppa4 mRNA level was normalized using GAPDH expression. A logarithmic scale of 2-ΔΔCT was used to represent the fold-change. **b** Western blot analysis was performed to access Dppa4 protein levels in 8 representative cases of colon cancer samples (T) and adjacent noncancerous tissues (N). **c** Dppa4 protein is higher in tumor tissues than in paired adjacent normal mucosa

The expression of Dppa4 in tumor tissue was examined and compared with paired normal colonic mucosa in 39 patient samples using real-time PCR with GAPDH as the internal reference. Among the 39 paired cases, 25 (64.1 %) colon cancer tissues showed at least a 2-fold increase in Dppa4 mRNA level compared with its adjacent non-cancerous mucosa (Fig. 1a). The average Dppa4 expression (ΔCt value) in the colon tumor group was 5.17 ± 0.37 whereas in the normal tissue group, it was 8.90 ± 0.55 (*P* < 0.01), indicating that the Dppa4 mRNA level was upregulated in cancer tissues than in paired normal mucosa. Subsequently, Western blotting showed a significant upregulation of Dppa4 protein expression in cancerous colon tissue compared with that of the corresponding normal tissue (2.44 ± 0.20 vs. 0.96 ± 0.06, respectively; *P* < 0.01, Student’s *t*-test) (Fig. 1b and c).

**Fig. 2:**
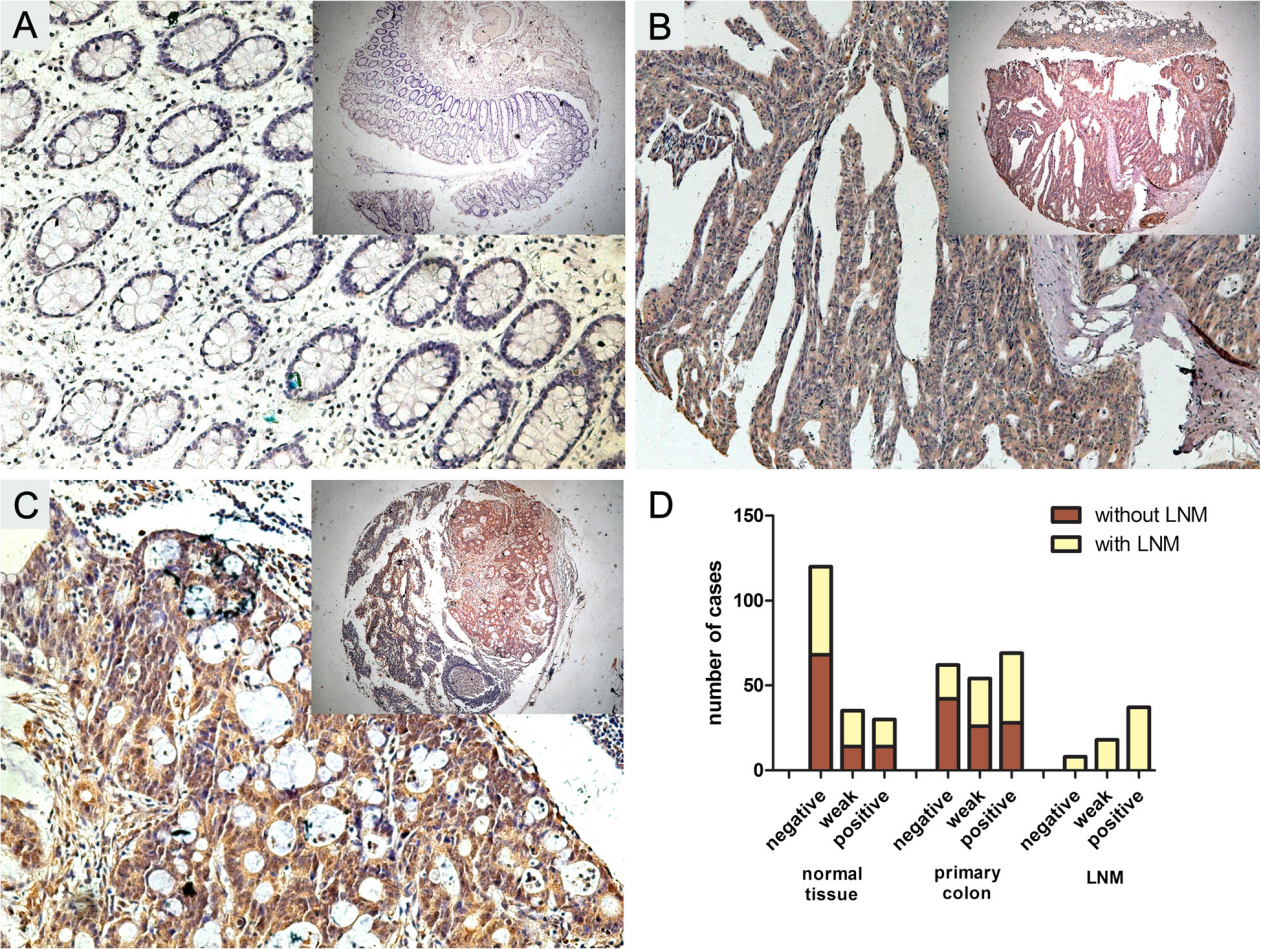
Immunohistochemical staining of Dppa4 expression in tissue microarrays. **a** Negative-Dppa4 expression in normal colonic epithelium and (**b**) Diffuse, intense Dppa4 staining in moderately and poorly-differentiated colon tumors; (**c**) colon cancer lymph node metastasis. Original magnification × 200 (×50 for inserts). (**d**) Dppa4 expression in 185 cases of paired normal colon tissue, primary colon cancer and 63 cases of lymph node metastasis (LNM). Dppa4 expression was significantly higher in LNM than in primary colon cancer and normal colon tissue. (*P* < 0.001)

The clinicopathologic significance of Dppa4 expression was further determined by immunohistochemical analysis on a tissue array containing 185 cases of primary colon cancer paired with normal tissue and 63 cases of available metastatic lymph nodes. As shown in Fig. 2, Dppa4 was prominently localized in the cytoplasm of the cancer cells and was prominently upregulated in 42.9 % (69 of 185) of primary cancer specimens and 58.7 % (37 of 63) of LNM, whereas the adjacent normal tissue was only 16.2 % (30 of 185), especially in cases with LNM. These results suggested a possible role of Dppa4 involved in the metastasis of colon cancer.

Association between Dppa4 immunoreactivity and clinicopathologic variables are summarized in Table 1. Dppa4 expression showed a positive result with the American Joint Committee on Cancer (AJCC) stage (*P* = 0.01), invasion depth (*P* = 0.028), nodal involvement (*P* = 0.012), distant metastasis (*P* = 0.003), and differentiation (*P* = 0.002).

**Table 1:** Association between clinicopathological features and DPPA4 or Ki-67 protein expression

	n	DPPA4 expression			*P* value
		Negative (n = 62) (%)	Weak (n = 54) (%)	Strong (n = 69) (%)	
Age, years (n,%)					0.423
<65	72	21 (33.9)	20 (37.0)	31 (44.9)	
≥65	113	41 (66.1)	34 (63.0)	38 (55.1)	
Gender (n,%)					0.519
Male	79	30 (48.4)	22 (40.7)	27 (39.1)	
Female	106	32 (51.6)	32 (59.3)	42 (60.9)	
Location (n,%)					0.650
Right	74	26 (41.9)	19 (35.2)	29 (42.0)	
Transverse	18	7 (11.3)	6 (11.1)	5 (7.2)	
Left	19	3 (4.8)	7 (13.0)	9 (13.0)	
Sigmoid colon	74	26 (41.9)	22 (40.7)	26 (37.7)	
T stage (n,%)					0.028*
T1	7	2 (3.2)	3 (5.6)	2 (2.9)	
T2	21	10 (16.1)	7 (13.0)	4 (5.8)	
T3	72	29 (46.8)	23 (42.6)	20 (29.0)	
T4	85	21 (33.9)	21 (38.9)	43 (62.3)	
N stage (n,%)					0.012*
N0	96	42 (67.7)	26 (48.1)	28 (40.6)	
N1	58	16 (25.8)	18 (33.3)	24 (34.8)	
N2	31	4 (6.5)	10 (18.5)	17 (24.6)	
M stage (n,%)					0.003*
M0	168	60 (96.8)	52 (96.3)	56 (81.2)	
M1	17	2 (3.2)	2 (3.7)	13 (18.8)	
AJCC stage (n,%)					0.010*
I	22	11 (17.7)	6 (11.1)	5 (7.2)	
II	71	29 (46.8)	20 (37.0)	22 (31.9)	
III	75	20 (32.3)	26 (48.1)	29 (42.0)	
IV	17	2 (3.2)	2 (3.7)	13 (18.8)	
Differentiation (n, %)					0.002*
High	90	41 (66.1)	27 (50.0)	22 (31.9)	
Moderate	68	17 (27.4)	20 (37.0)	31 (44.9)	
Low	27	4 (6.5)	7 (13.0)	16 (23.2)	
Vascular invasion (n, %)					0.420
Yes	173	60 (96.8)	50 (92.6)	63 (91.3)	
No	12	2 (3.2)	4 (7.4)	6 (8.7)	
Ki-67 index (n, %)					0.071
Negative	36	13 (21.0)	15 (27.8)	8 (11.6)	
Positive	149	49 (79.0)	39 (72.2)	61 (88.4)	

*P < 0.05 indicates a significant association among the variables

### High Dppa4 expression associated with poor clinical outcome in human colon cancer

**Fig. 3:**
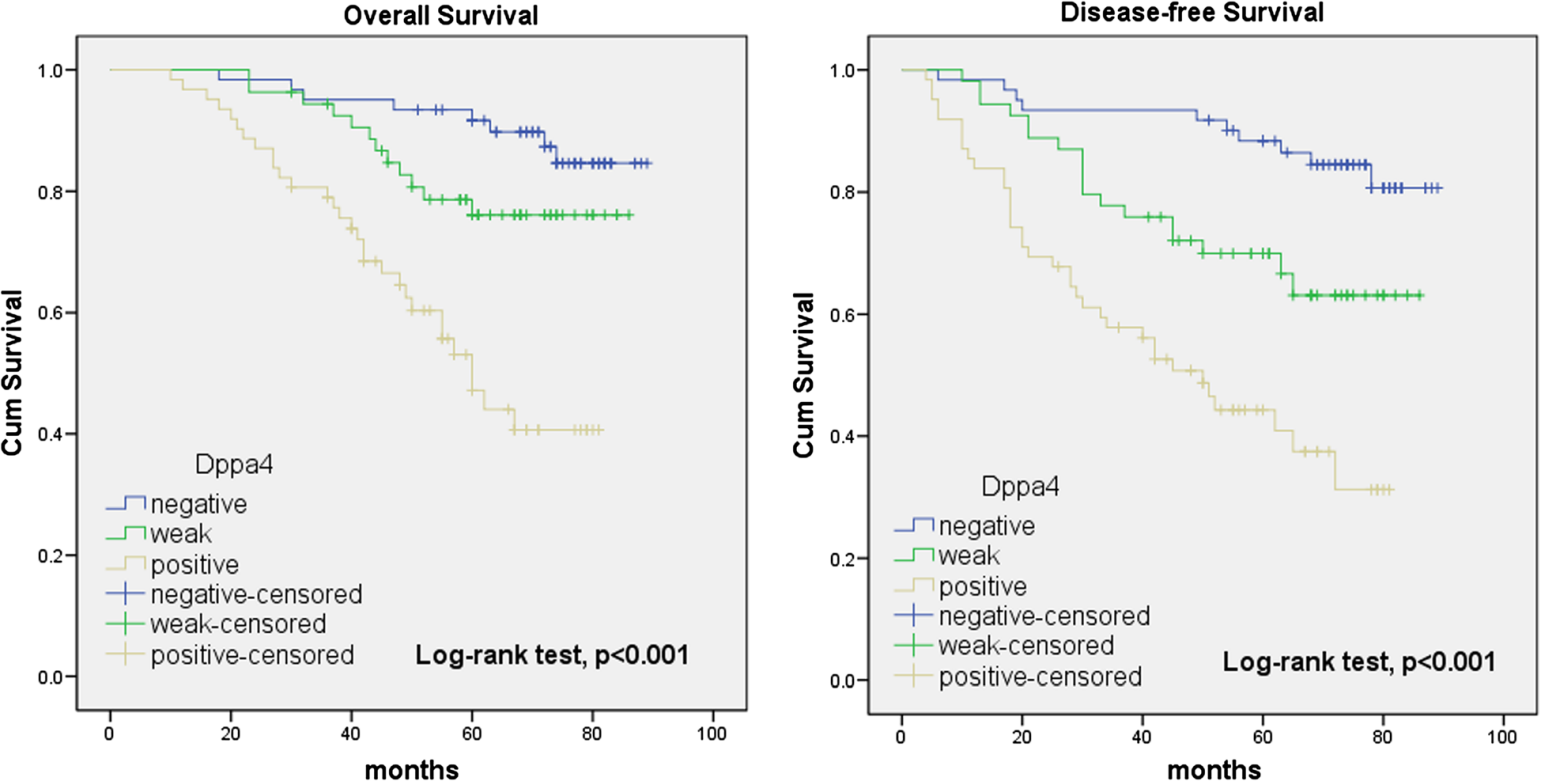
Kaplan-Meier survival analysis of 5-year OS and DFS in 177 patients with colon cancer. Dppa4 expression was dramatically correlated with poor OS and DFS

To assess the predictive role of Dppa4 for distant metastasis, Kaplan-Meier curves with a log-rank test for OS and DFS was undertaken in 177 patients who accepted radical colectomy. There was a significant difference between Dppa4-positive and Dppa4-negative groups after the surgery (Fig. 3). Patients with Dppa4-positive tumors subsequently developed metastasis or local recurrence than those with Dppa4-negative tumors (*P* < 0.01) [Dppa4-positive: 54 of 116 patients (46.6 %); Dppa4-negative: 10 of 61 (16.4 %)]. When compared with patients with Dppa4-negative tumors, the DFS rate was significantly lower in patients who had Dppa4-positive primary tumors (Fig. 3, log-rank test, *P* < 0.001). Kaplan-Meier analysis also revealed that Dppa4 expression was significantly relevant to OS of colon cancer patients (log-rank test, *P* < 0.001, Fig. 3). Patients with Dppa4-positive tumors had a significantly lower 5-year OS than those with Dppa4-negative tumors (57.8 % vs. 86.9 %).

In univariate analysis, patients with Dppa4-positively stained colon tumors had an obviously lower DFS and OS than those with Dppa4-negative tumors [DFS, HR 6.118 (95 % CI 3.004–12.462); OS, HR 6.348 (95 % CI 2.875–14.014)] (Table 2). In addition, pT stage, pN stage, AJCC stage, tumor differentiation, vascular invasion and Ki-67 index was correlated with OS and DFS. In multivariate analysis with clinicopathologic parameters, the expression of Dppa4 was found to be an independent prognostic marker to predict tumor recurrence (Table 3).

**Table 2:** Univariate Cox proportional hazards model for overall survival (OS) and disease-free survival (DFS)

	DFS			OS		
	HR	95 % CI	*P*-value	HR	95 % CI	*P*-value
Age, years						
<65	—			—		
≥65	1.100	0.660–1.834	0.714	0.934	0.530–1.644	0.812
Gender						
Male						
Female	1.013	0.617–1.662	0.960	1.546	0.860–2.776	0.145
Tumor location						
Right	—			—		
Transverse	0.816	0.309–2.155	0.681	0.789	0.267–2.334	0.669
Left	1.392	0.619–3.130	0.423	1.291	0.512–3.252	0.589
Sigmoid colon	1.250	0.718–2.176	0.431	1.145	0.614–2.135	0.671
T stage						
T1	0.494	0.119–2.049	0.331	0.988	0.299–3.259	0.984
T2	0.203	0.063–0.660	0.008*	0.394	0.138–1.130	0.083
T3	0.484	0.284–0.827	0.008*	0.520	0.280–0.966	0.039*
T4	—			—		
N stage						
N0	—			—		
N1	5.887	3.025–11.456	<0.001*	4.157	2.009–8.602	<0.001*
N2	15.914	7.781–32.545	<0.001*	13.193	6.149–28.307	<0.001*
AJCC stage						
I	—			—		
II	1.108	0.305–4.027	0.877	0.667	0.201–2.215	0.508
III	6.823	2.097–22.199	0.001*	3.401	1.184–9.771	0.023*
IV	49.185	12.615–191.764	<0.001*	40.074	11.257–142.668	<0.001*
Differentiation						
High	—			—		
Moderate	1.315	0.750–2.306	0.340	1.458	0.764–2.780	0.253
Low	3.577	1.885–6.786	<0.001*	4.358	2.140–8.872	<0.001*
Vascular invasion						
Yes	4.900	2.469–9.721	<0.001*	4.638	2.152–9.997	<0.001*
No	—			—		
Ki-67 index						
Negative	—			—		
Weak	1.931	0.748–4.985	0.174	0.794	0.308–2.051	0.634
Positive	2.667	1.203–5.913	0.016*	1.175	0.592–2.335	0.645
DPPA4 expression						
Negative	—			—		
Weak	2.598	1.194–5.653	0.016*	2.117	0.862–5.196	0.102
Positive	6.118	3.004–12.462	<0.001*	6.348	2.875–14.014	<0.001*

*P < 0.05 indicates a significant association among the variables

**Table 3:** Multivariate Cox proportional hazards model for OS and DFS

	DFS			OS		
Variable	HR	95 % CI	*P*-value	HR	95 % CI	*P*-value
DPPA4 expression	2.826	1.920–4.159	<0.001*	2.664	1.721–4.123	<0.001*
T stage	1.692	1.128–2.538	0.011*	3.989	1.754–9.073	0.001*
N stage	3.709	2.053–6.699	<0.001*	3.313	1.773–6.193	<0.001*
M stage	4.352	1.303–14.542	0.017*	8.000	2.393–26.745	0.001*
Ki-67 expression	1.226	0.790–1.904	0.363	1.570	0.846–2.914	0.153

*P < 0.05 indicates a significant association among the variables

### Knockdown of Dppa4 expression inhibits the proliferation of colon cancer cells

**Fig. 4:**
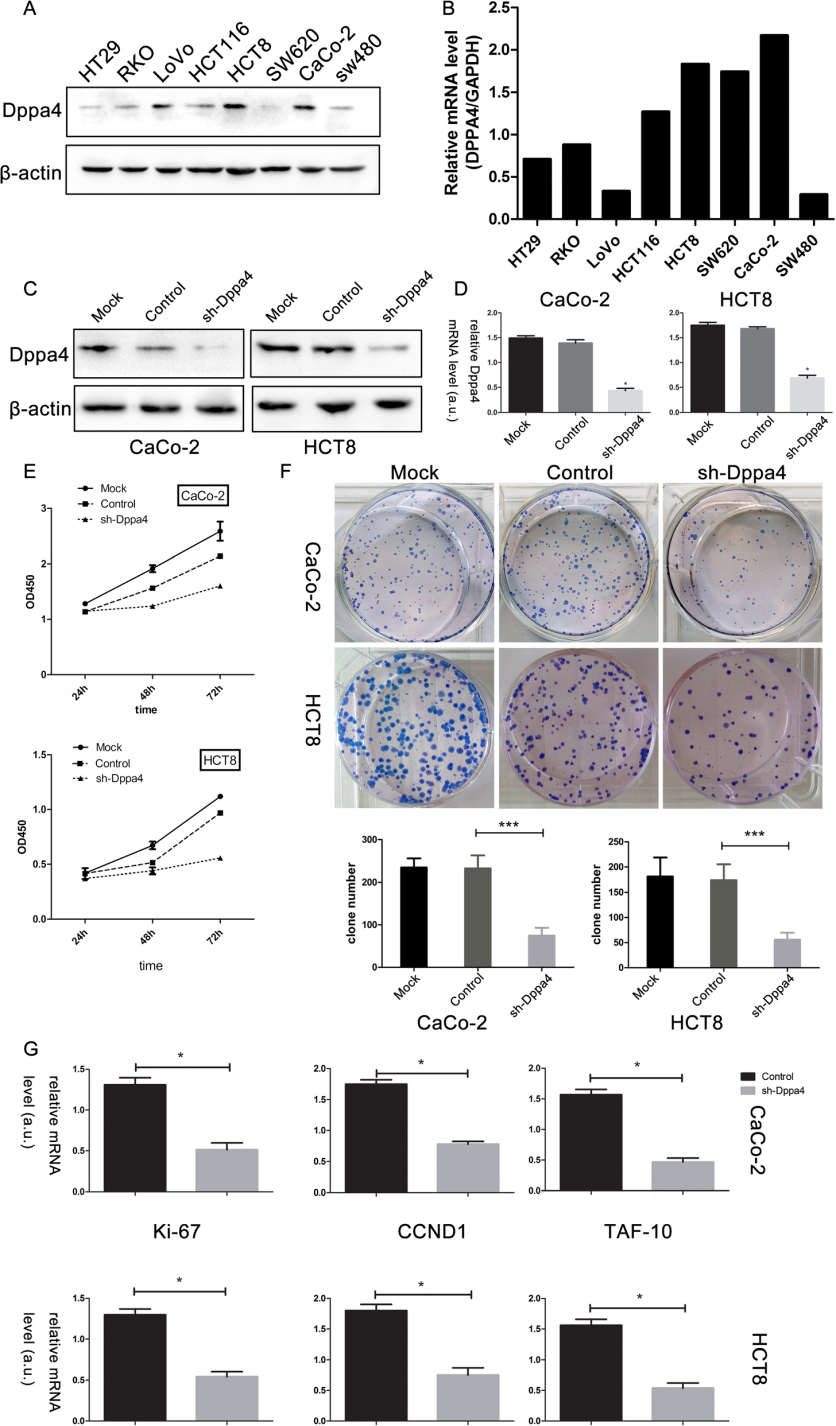
Dppa4 expression in cell lines and its knockdown inhibits cancer cell proliferation. **a** Dppa4 protein levels and (**b**) mRNA levels in 8 colorectal cancer cell lines. **c** Western blot analysis, and (**d**) real-time PCR analysis of Dppa4 expression in stable knockdown CaCo2 and HCT8 cell lines. **e** Effects of Dppa4 knockdown on proliferation was evaluated by Cell Counting Kit-8 assays and (**f**) plate colony formation assays. **g** Real-time PCR analysis of proliferation related genes and G1/S transition genes. (* *P* < 0.05, *** *P* < 0.01)

To examine the effect of Dppa4 on colon cancer, a series of functional experiments related to cell proliferation were performed. Quantification by qRT-PCR and Western blot denoted that Dppa4 expression level was higher in HCT8 and CaCo2 than other cell lines (Fig. 4a, b). RNA interference (RNAi) technology was carried out to access the role of Dppa4 in colon cancer cells. The efficiency of shRNA-mediated knockdown in HCT8 or CaCo2 cells was more than 60 % (Fig. 4c, d). CCK-8 assays showed a significant inhibition of cell growth in the Dppa4 shRNA group compared with the control groups at 24-, 48-, and 72-h time points (Fig. 4e). Colony formation assay showed that Dppa4 knockdown inhibited cancer cell colonogenicity compared with control groups (Fig. 4f).

The mRNA level of proliferation-related gene ki-67 and G1/S transition related genes, CCND1 and TAF10 was evaluated to confirm the effect of Dppa4 knockdown on cancer cell proliferation. Compared with the control cells, Dppa4 shRNA group showed a lower ki-67, CCND1, and TAF10 expression (Fig. 4g).

## Discussion

It has been noted for years that cancer cells and embryonic stem (ES) cells share some common characteristics with respect to self-renewal, proliferation, and indefinite growth [18]. The possible explanation for this phenomenon is that these cells share some common genes. It had been reported that many important stem cell markers such as Oct-4, Sox-2, and Nanog are highly expressed in cancer tissues and cancer cells [19, 20].

 In this study, we explored the expression pattern of DPPA4 in colon cancer samples and cell lines. Our results showed for the first time that (1) over-expression of Dppa4 was significantly associated with unfavorable clinicopathological variables and Dppa4 might be used as a novel prognostic marker for colorectal cancer. (2) DPPA4 expression level was associated with cancer progression and proliferation.

 Dppa gene family is a cluster of five genes whose developmental expression patterns are similar to those of Oct4 [21]. The expression of Dppa4 is restricted during early developmental stages and in germ cells including primordial germ cells, gonads, and testis [22]. It has a putative DNA-binding domain named SAP (Scaffold attachment factor A/B, Acinus, and PIAS), which is found in SAF-A/B, poly (ADP-ribose) polymerase, Ku70, PIAS, and Acinus and is thought to be involved in chromosomal organization including RNA processing, DNA repair, and apoptotic degradation of chromatin [23]. It has also been reported that Dppa4 is a nuclear factor that is associated with active chromatin and also regulates differentiation of ES cells into a primitive ectoderm lineage [24]. The structure and functions of Dppa4 in biology process indicate the possible mechanism of Dppa4 in cancer progression and it might be a potential therapeutic target. Dppa4 re-expression has been found in tissue samples of colon, prostate, and bladder carcinomas as well as cancer cell lines HT-29, Caco-2, HT-1376, LNCaP, and HepG2 [9]. Recently, Dppa4 was identified as a novel family of pluripotency-related oncogene [13]. However, the prognostic potential role of Dppa4 in human colon cancer and its potential role in the pathogenesis and progression of colon cancer still remain unclear.

Our results showed that Dppa4 expression was up-regulated in cancerous colonic tissues compared with normal epithelium at the transcriptional as well as translational level. Overexpression of Dppa4 was associated with cancer progression and metastasis. Individuals with Dppa4-positively stained tumors experienced shorter OS and DFS, indicating Dppa4 as a novel prognostic biomarker of colon cancer outcome. Finally, by univariate and multivariate Cox model analyses, we confirmed Dppa4 to be an independent prognostic factor for DFS and OS in colon cancer.

To further understand the role of Dppa4 in tumorigenesis, shRNA was used to knockdown the expression of Dppa4 in HCT-8 and CaCo2 cells. Then CCK8 and colony formation assays were implemented, which revealed that knockdown of Dppa4 inhibited cell proliferation activity. The expression of ki-67, which is considered as a proliferation-related gene, showed obvious slumps in Dppa4 knockdown cells than control groups. It is reported that overexpression of Dppa4 could alter the levels of several major components in p53 signaling pathway, including E2F1, MDM2, CDKN1A (p21), TRP63, BAX, APAF1, and GADD45A, and DPPA4 can induce cell proliferation through genes (including CCND1, TAF10 and E2F1, which is considered as putative G1/S related genes) related to regulation of the G1/S transition [13]. We examined the expression of CCND1 and TAF10 by RT-PCR, and found that mRNA levels of both the genes were significantly downregulated in Dppa4 knockdown cells, indicating that Dppa4 suppressed cancer cell proliferation through the regulation of G1/S transition, demonstrating that Dppa4 is a potential therapeutic target for colon cancer.

Study limitations include the small scale of investigated patients with relatively short follow-up time, lack of overexpressed cancer cell lines and *in vitro* experiment setting. Further studies are needed to explore the role of increased Dppa4 expression in the early stage of colon cancer and the mechanism of Dppa4 deregulation in colon cancer and its relation with p53 pathway and the regulation of cell cycle.

## Conclusions

In summary, the present study identified that the Dppa4 mRNA and protein expression was upregulated in the colon cancer samples. Dppa4 overexpression was associated with poor OS and DFS of colon cancer patients. Dppa4 was also a significant independent prognostic factor for colon cancer. Furthermore, knockdown of Dppa4 inhibited cell proliferation through the regulation to G1/S transition. Therefore, Dppa4 is associated with malignant transformation of colon cancer and is a potential target for cancer prevention and treatment.
